# Disruption of the histone acetyltransferase GCN5 and the transcriptional coactivator ADA2b affect trichome density in *Arabidopsis thaliana*

**DOI:** 10.17912/micropub.biology.000174

**Published:** 2019-10-17

**Authors:** Jenna Kotak, Ashley Kendig, Kelly Cann, Joshua Shaffer, Amy T Hark, Elizabeth R McCain

**Affiliations:** 1 Biology Department, Muhlenberg College, Allentown, PA 18104, USA; 2 Molecular Biology, Cell Biology, and Biochemistry Department, Brown University, Providence, RI 02912, USA; 3 Molecular, Cell, and Developmental Biology, University of California at Santa Cruz, Santa Cruz, CA 95064, USA

**Figure 1:**
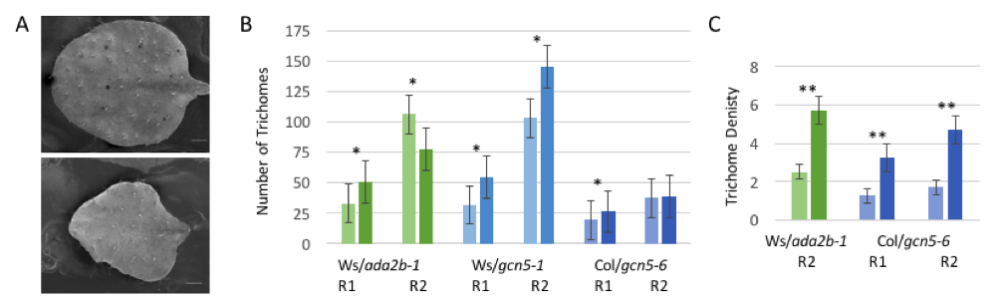
The number and density of rosette leaf trichomes is increased in *ada2b* and *gcn5* *Arabidopsis* mutants. A. Scanning electron micrographs of Ws-2 (top) and *ada2b-1* (bottom) second rosette leaves. Bar = 0.71 mm. B. Number of trichomes (y-axis) on first pair of leaves (R1) and second rosette leaves (R2) on wildtype (Ws-2 or Col-0) and mutant plant lines as shown, with darker bars at the right within each grouping representing the homozygous mutant samples. Between 7-32 leaves were examined for each genotype, with an asterisk * denoting a significant difference between wildtype and mutant (p<0.05). Error bars show standard error. C. Trichome density (average number of trichomes/mm^2 ^leaf surface area; y-axis). Between five and nine leaves were examined for each genotype, with darker bars at the right within each grouping representing the mutant samples. A double asterisk ** denotes a significant difference between wildtype (Ws-2 or Col-0) and mutant (p<0.005). Error bars show standard error.

## Description

GCN5 is a histone acetyltransferase that is well-conserved in eukaryotic species and has been shown to play roles in global histone modification and gene expression. The transcriptional coactivator ADA2 works with GCN5 and other regulators in these processes (Candau *et al.* 1997; Grant *et al.* 1997); in *Arabidopsis thaliana* the functional paralog is referred to as ADA2b (Hark *et al.* 2009). Pleiotropic phenotypes of plants with T-DNA insertions at these loci were first reported fifteen years ago (Vlachonasios *et al.* 2003; Bertrand *et al.* 2003). Since then, we and others have sought to understand the roles of GCN5 and ADA2b in specific developmental pathways. We recently reported that rosette leaf trichomes display altered ploidy and branching morphology in *gcn5* and *ada2b* mutants (Kotak *et al.* 2018). This suggests that these chromatin factors impact later steps in trichome morphogenesis (Hulskamp 2004).

The data reported here suggest that GCN5 and ADA2b also affect trichome initiation. Using scanning electron microscopy to visualize trichomes on rosette leaves (Fig. 1A), we determined that the number of trichomes on the first true leaf is increased in *ada2b-1* as well as *gcn5* mutant backgrounds (Fig. 1B). On the second rosette leaf, a similar effect was seen in plants homozygous for the hypomorphic *gcn5-1* allele while in *gcn5-6* where the catalytic domain of GCN5 is disrupted, there was no distinguishable difference in trichome number (Fig. 1B). When looking at the second rosette leaf in *ada2b-1* plants, trichome number was increased in the mutant background. However, counting total number of trichomes does not take into account leaf size. In cases in which we did not detect an obvious increase in total number of trichomes, there is a significant increase in trichome density (Fig. 1C).

## Reagents

*Arabidopsis thaliana* plants were germinated in soil and grown at 22°C under continuous light conditions (140 µmoles/m^2^/sec). Plants were watered once a week with Hoagland’s solution and a second time weekly (or as needed) with deionized water. Plants were genotyped via PCR and leaves were prepared for scanning electron microscopy (SEM) as described previously (Kotak *et al.* 2018). Each leaf was examined under the SEM and the total number of trichomes per leaf was counted. ImageJ was used to measure each leaf’s surface area from an SEM image and the trichome density was then calculated. Statistical analyses of the data were made using Student’s t-test.
